# Upstream-Downstream Joint Carbon Reduction Strategies Based on Low-Carbon Promotion

**DOI:** 10.3390/ijerph15071351

**Published:** 2018-06-27

**Authors:** Xiqiang Xia, Junhu Ruan, Zhiru Juan, Yan Shi, Xuping Wang, Felix T. S. Chan

**Affiliations:** 1 Business School, Zhengzhou University, Zhengzhou 450001, China; xqxia@zzu.edu.cn; 2 Department of Industrial and Systems Engineering, The Hong Kong Polytechnic University, Hung Hom, Hong Kong, China; f.chan@polyu.edu.hk; 3 School of Economics and Management, Huaiyin Normal University, Huaian 223300, China; juanzhiru@163.com; 4 General Education Center, Tokai University, Kumamoto 862-8652, Japan; yshi@ktmail.tokai-u.jp; 5 Institute of Systems Engineering, Dalian University of Technology, Dalian 116024, China; wxp@dlut.edu.cn

**Keywords:** carbon reduction, low-carbon promotion, differential game model

## Abstract

A differential game model is established to analyze the impact of emissions reduction efforts and low-carbon product promotion on the reduction strategies of low-carbon product manufacturers (subsequently referred to as manufacturers) and the retailers of such products in a dynamic environment. Based on this model, changes in emissions reduction efforts and promotional efforts are comparatively analyzed under three scenarios (retailers bearing the promotional cost, manufacturers bearing the promotional cost, and centralized decision-making). The results are as follows: (1) the trajectory of carbon emissions reduction per product unit is the highest when the supply chain is under centralized decision-making, followed by when manufacturers bear the promotional cost, and lastly when retailers bear the cost; (2) when manufacturers bear the promotional cost, the market demand, emissions reduction effort, and promotional effort are higher, although the unit retail price is higher than when retailers bear the promotional cost; and (3) under centralized decision-making, the unit retail price is the lowest; however, sales volume, the emissions reduction effort, and the promotional effort are all higher than those in the other scenarios.

## 1. Introduction

The United Nations Intergovernmental Panel on Climate Change (IPCC) noted that despite being a natural change, global warming is largely attributable to human activities, particularly to carbon dioxide emissions from human activity. The impact of household consumption on environmental quality is approximately 30–40%. However, obviously, putting a complete stop to human consumption is impossible. Therefore, the key to solving the problem is to change the fundamental consumption pattern to low-carbon consumption [1,2,3,4]. In promoting the development of low-carbon consumption, it is essential that consumers have a clear understanding of such consumption. To encourage low-carbon consumption, the United States and developed countries in Europe have used various communications media to publicize such consumption and improve the environmental awareness of citizens. For example, to change consumption patterns, the British media were used to introduce a green consumption action plan to promote environmentally friendly consumer behavior, such as choosing energy-saving products and recycling waste. The Japanese government organized “Reduce, Reuse, Recycle” (3R)-themed public activities to promote green packaging and waste recycling. France launched a waste reduction program that called on the public to reduce its use and waste of disposable office supplies. China has also adopted measures to promote low-carbon consumption. For example, in 2013, National Low-carbon Day was established by the State Council. In response to the promotion of low-carbon consumption, an interest in low-carbon consumption has gradually formed among consumers, who have become willing to buy low-carbon products and pay more for them.

Under the Chinese government’s low-carbon economy initiative, companies are paying increasing attention to energy saving, emissions reduction, and the production of low-carbon products. Although manufacturer efforts play an important role in energy saving and emissions reduction, to enable consumers to fully understand low-carbon products and their role in emissions reduction, promotional efforts are required. To increase consumer understanding of corporate efforts to reduce carbon emissions and of low-carbon consumption as well as to increase market demand, manufacturers have begun to enhance their promotion of low-carbon products. However, the effects of promoting low-carbon products require more than one cycle to become evident. In addition, low-carbon products can be advertised by either retailers or manufacturers (which is primarily manifested in the different forms of bearing the promotional cost). Therefore, adopting a long-term and dynamic perspective, this paper examines the emissions reduction efforts and promotional issues faced by manufacturers and retailers when low-carbon promotional costs are covered differently, and establishes a decision-making basis for the business strategy development of low-carbon product manufacturers.

Research conducted on consumer behavior and changes in low-carbon product consumption has mainly focused on two aspects: (1) the impact of consumer preferences on the low-carbon supply chain and (2) the role of promotion in the low-carbon supply chain. A number of studies have addressed the impact of consumer preferences on low-carbon supply chains. Wei et al. [2] investigated the low-carbon consumption habits of Chinese citizens through questionnaires and analyzed behavioral factors that affect low-carbon consumption. Li et al. [3] observed that in the consumer process, consumers gradually become concerned about environmental protection performance and the environmental records of manufacturers. Du et al. [5] assumed decision-makers in an emission-concerned supply chain are emission-sensitive, formulated a corresponding demand function and cost function, and investigated the decision-making of each member in the supply chain. Wang et al. [6] assumed consumers are environment conscious and observed the impact of market low-carbon preference on the performance of a two-echelon supply chain. As we can see, some consumer preferences such as emission-sensitive and cooperation friendly have been well recognized, but researchers may have different opinions on some specific preferences. For example, [1,6], respectively, considered the competitive and non-collaborative members in the emissions reduction of supply chains. In this work, we considered both decentralized and centralized scenarios.

Researchers have also investigated the impact of low-carbon promotion on various supply chains. Ji et al. [7] discussed the emission reduction strategies of an O2O (Online To Offline) retail supply chain under three low-carbon cases: without cap-and trade regulation, cap-and-trade regulation based on grandfathering mechanism, and cap-and-trade regulation based on benchmarking mechanism. Bai et al. [8] considered the promotional effort, product selling price, and the sustainable level to study a two-echelon perishable supply chain under carbon cap-and trade regulation. Shao et al. [9] analyzed the impact of subsidy incentive and price discount incentive on the adoption of low-carbon products in the vehicle market consisting of the government, manufacturers, and consumers. Cao et al. [10] observed the impacts of cap-and-trade policy (CTP) and low carbon subsidy policy (LCSP) on the carbon emission reduction level of manufacturers and found that higher carbon trading price results in higher emissions reduction level.

In sum, carbon emissions reduction has become a popular research topic in China and abroad, and an abundance of literature is available, including articles [1,2,3,4,5,6] on the impact of the low-carbon preferences of consumers on the low-carbon supply chain and articles [7,8,9,10] that analyze the impact of low-carbon promotion on low-carbon industry development. However, few studies consider the question of joint emissions reductions by manufacturers and retailers. Although [6] examines joint emissions reduction in the supply chain, the research is based on the assumption that the marginal returns are unchanged. In fact, the impact of emissions reduction efforts and low-carbon promotion on the unit wholesale price and the retail price varies. Therefore, based on the literature, this paper constructs a differential game model for joint emissions reduction by manufacturers and retailers under the condition of changing wholesale and retail prices. In addition, the paper compares the impact of various approaches for covering promotional costs in the low-carbon supply chain to establish a basis for decision-making by manufacturers and retailers.

The rest of the work is organized as follows. In Section 2, we make clear the focusing problem and corresponding assumptions. In Section 3, three above scenarios in the low-carbon supply chain are analyzed with corresponding results and insights. Simulations are made in Section 4 to verify our theoretical analysis results. Section 5 concludes the work.

## 2. Problem Description and Assumptions

### 2.1. Problem Description

This paper constructs a differential game model to simulate low-carbon emissions reduction efforts and promotional efforts to support low-carbon products by manufacturers and retailers. Using the model, the paper first studies the issue of joint emissions reduction by a joint supply chain under two scenarios: (1) the low-carbon products’ promotional cost is borne by the manufacturers; or (2) by the retailers; (3) The paper also considers low-carbon emissions reduction in the supply chain under centralized decision-making. The decision-making process of each scenario is as follows:

Scenario 1: The retailers bear the promotion cost. The manufacturers bear the cost of emissions reduction effort, and the retailers bear the cost of promotional effort. The manufacturers sell products to the retailers at the whole price, and then, the retailers sell products to consumers at the retail price. However, the retail price depends on the whole price that depends on the degree of emissions reduction effort and the degree of promotion effort. Thus, the decision-making process can be described as follows: Firstly, the manufacturers and retailers individually determine the emissions reduction efforts and promotion efforts. Then, the manufacturers determine the unit wholesale price according to emissions reduction efforts and promotion efforts. Finally, the retailers determine the unit retail price according to the unit wholesale price.

Scenario 2: The manufacturers bear the promotion cost. The cost of emissions reduction efforts and the cost of promotional efforts are only borne by the manufacturers. The manufacturers sell products to the retailers at the whole price, and then, the retailers sell products to consumers at the retail price. However, the retail price depends on the whole price, and the whole price depends on the degree of emissions reduction efforts and the degree of promotion efforts. Thus, the decision-making process can be described as follows: The manufacturers firstly determine emissions reduction efforts and promotion efforts. Then, the manufacturers determine the unit wholesale price according to emissions reduction efforts and promotion efforts. Finally, the retailers determine the unit retail price according to the unit wholesale price.

Scenario 3: Centralized decision-making. There is no retailer in scenario 3, i.e., there is only the manufacturer. Thus, the decision maker is the manufacturer. The manufacturer directly sells products to consumers. The retail price depends on the degree of emissions reduction efforts and the degree of promotion efforts. Thus, the decision-making process can be described as follows: The manufacturers firstly determine emissions reduction efforts and promotion efforts. Then, the manufactures determine the unit price according to emissions reduction efforts and promotion efforts.

### 2.2. Symbol Description

Superscript R: the retailer bears the low-carbon product promotional costs;

Superscript M: the manufacturer bears the low-carbon product promotional costs;

Superscript C: centralized decision-making is used in the low-carbon supply chain;

$\tau^{i}(t)$: amount of emissions reduction per product unit from the manufacturer under scenario i at time t, where i∈{R, M, C};

$E^{i}{}_{M}(t)$: degree of emissions reduction effort at time t under scenario i, where i∈{R, M, C};

$E_{R}^{i}(t)$: degree of low-carbon product promotion effort at time t under scenario i, where i∈{R, M, C}

### 2.3. Model Assumptions

(1) According to [11,12], the relationship between the cost of the emissions reduction effort for low-carbon products and the level of effort in emissions reduction, $E_{M}^{i}(t)$, is as follows. The cost of the emissions reduction effort is $\frac{C_{m}E_{M}^{i}{\left(t\right)}^{2}}{2}$, where $C_{m}$ ($C_{m}>0$) indicates the coefficient of the cost of the emissions reduction effort. The relationship between the cost of the promotional effort for low-carbon products and the degree of the promotional effort, $E_{R}^{i}(t)$, is as follows. The cost of the promotional effort $\frac{C_{R}E_{R}^{i}{\left(t\right)}^{2}}{2}$, where $C_{R}$ ($C_{R}>0$), indicates the coefficient of the cost of the promotional effort.
(2) The unit product emissions reduction is related to the degree of emissions reduction effort of the manufacturer and is a dynamic process. The differential equation, Equation (1), shows the process of change in product unit emissions reduction:
  (1) $$\frac{d\tau (t)}{dt}=\gamma E_{M}^{}(t)-\delta \tau (t)$$
  In the equation, τ(t) is the amount of emissions reduction per unit of product at time *t*. For the initial emissions reduction $\tau (0)=\tau_{0}\geq 0$, γ indicates the extent of the impact of the emissions reduction effort on the emissions reductions per unit of product and δ(δ>0) indicates the attenuation coefficient of emissions reduction due to aging equipment without any emissions reduction effort. Similar usage of (1) is visible in [6,13].
(3) The demand function is as follows:
  (2) $$D(p(t))=Q-\alpha p(t)+\beta E_{R}(t)+\theta \tau (t)$$
  In the equation, Q represents the potential demand for low-carbon products in the market, α(α>0), *α* indicates the reaction coefficient of consumers to unit retail price p(t), β(β>0) indicates the reaction coefficient of consumers to the extent of emissions reduction efforts in low-carbon products, and θ(θ>0) indicates the reaction coefficient of consumers to the extent of promotional efforts to support low-carbon products. Similar usage of (2) is visible [14].
(4) According to [15,16], manufacturers and retailers have the same discount rate ρ, and ρ>0. The goal of both entities is to maximize individual profit within the infinite interval.

## 3. Model Solution

### 3.1. Under Decentralized Decision-Making

#### 3.1.1. When Retailers Bear the Promotional Cost of Low-Carbon Products

The manufacturer’s objective function is as follows:

(3) $$\underset{w(t),E_{M}(t)}{\max}J_{M}^{R}=\int_{0}^{\infty }e^{-\rho t}[(w(t)-c)(Q-\alpha p(t)+\beta E_{R}(t)+\theta \tau (t))-\frac{C_{M}E_{M}{\left(t\right)}^{2}}{2}]d(t)$$

The retailer’s objective function is as follows:

(4) $$\underset{p(t),E_{R}(t)}{\max}J_{R}^{R}=\int_{0}^{\infty }e^{-\rho t}[(p(t)-w(t))(Q-\alpha p(t)+\beta E_{R}(t)+\theta \tau (t))-\frac{C_{R}E_{R}{\left(t\right)}^{2}}{2}]d(t)$$

According to the optimal control principle, the Hamilton functions for the manufacturer and the retailer can be obtained. For convenience, time *t* is omitted from the following expressions.

(5) $$H_{M}^{R}(w,\;E_{M},\;u_{M}^{R})=(w-c)(Q-\alpha p+\beta E_{R}+\theta \tau )-\frac{C_{M}E_{M}{}^{2}}{2}+u_{M}^{R}(\gamma E_{M}-\delta \tau )$$

(6) $$H_{R}^{R}(p,\;E_{R},\;u_{R}^{R})=(p-w)(Q-\alpha p+\beta E_{R}+\theta \tau )-\frac{C_{R}E_{R}{}^{2}}{2}+u_{R}^{R}(\gamma E_{M}-\delta \tau )$$

To simplify the solution, we first have Lemma 1.

**Lemma** **1.** *Equation (6) on* $p,\;E_{R}$ *is a concave function, and after substituting the solution obtained from Equation (6) into Equation (5), Equation (5) on* $w,\;E_{M}$ *is a concave function.*

**Proof.**  The Hessian matrix in Equation (6) is $H=\left[\begin{array}{cc} -2\alpha & 0\\ 0 & -C_{R} \end{array}\right]$, and from $H=\left|\begin{array}{cc} -2\alpha & 0\\ 0 & -C_{R} \end{array}\right|=2\alpha C_{R}>0$ and −2α<0, we can see that the matrix is negative semi-definite and the objective function is a concave function. Therefore, Equation (6) is a concave function on $p,\;E_{R}$, and its maximum value is reached at the zero point of its first derivative. Similarly, it can be demonstrated that Equation (5) on $w,\;E_{M}$ is a concave function. □

**Proposition** **1.** *The steady state of the system is as follows:*

(7) $$\left(\bar{\tau_{M}^{R}},\;\bar{u_{m}^{R}})=(\frac{\theta C_{R}\gamma^{2}(Q-\alpha c)}{\delta C_{M}A(\gamma +\delta )-C_{R}\theta^{2}\gamma^{2}},\;\frac{\delta \theta C_{R}C_{M}(Q-\alpha c)}{\delta C_{M}A(\gamma +\delta )-C_{R}\theta^{2}\gamma^{2}}\right)$$

*The retailer’s optimal unit retail price, degree of promotional effort, and sales volume are as follows, respectively:*

(8) $$P^{R*}=\frac{3C_{R}Q+c(\alpha C_{R}-\beta^{2})}{A}+\frac{3\theta C_{R}}{A}[(\tau_{0}-\bar{\tau_{M}^{R}})e^{-\frac{\varphi_{R}-C_{M}A\gamma }{2C_{M}A}t}+\bar{\tau_{M}^{R}}]$$

(9) $$E_{R}^{R*}=\frac{\beta (Q-\alpha c)}{A}+\frac{\beta \theta }{A}[(\tau_{0}-\bar{\tau_{M}^{R}})e^{-\frac{\varphi_{R}-C_{M}A\gamma }{2C_{M}A}t}+\bar{\tau_{M}^{R}}]$$

(10) $$q^{R*}=\frac{\alpha C_{R}(Q-\alpha c)}{A}+\frac{\alpha \theta C_{R}}{A}[(\tau_{0}-\bar{\tau_{M}^{R}})e^{-\frac{\varphi_{R}-C_{M}A\gamma }{2C_{M}A}t}+\bar{\tau_{M}^{R}}]$$

*The manufacturer’s optimal unit wholesale price and emissions reduction efforts are as follows, respectively:*

(11) $$w^{R*}=\frac{2C_{R}Q+c(2\alpha C_{R}-\beta^{2})}{A}+\frac{2\theta C_{R}}{A}[(\tau_{0}-\bar{\tau_{M}^{R}})e^{-\frac{\varphi_{R}-C_{M}A\gamma }{2C_{M}A}t}+\bar{\tau_{M}^{R}}]$$

(12) $$E_{M}^{R*}=\frac{\left(2\delta C_{M}A+C_{M}A\gamma -\varphi_{R})(\tau_{0}-\bar{\tau_{M}^{R}}\right)}{2C_{M}A\gamma }e^{-\frac{\varphi_{R}-C_{M}A\gamma }{2C_{M}A}t}+\frac{\delta }{\gamma }\bar{\tau_{M}^{R}}$$

*The optimal trajectory for the manufacturer’s product emissions reduction is as follows:*

(13) $$\tau^{R}=(\tau_{0}-\bar{\tau_{M}^{R}})e^{-\frac{\varphi_{R}-C_{M}A\gamma }{2C_{M}A}t}+\bar{\tau_{M}^{R}}$$

*where*$A=4\alpha C_{R}-\beta^{2},\;\varphi_{R}=\sqrt{C_{M}A[C_{M}A{\left(\gamma +2\delta \right)}^{2}-4C_{R}\gamma^{2}\theta^{2}]}$.

**Proof.**  From Lemma 1, we can see that by differentiating Equation (6) with respect to $p,\;E_{R}$ Equation set (14) can be obtained.

(14) $$\left\{ \begin{array}{l} \frac{\partial H_{R}^{R}}{\partial p}=-2\alpha p+Q+\beta E_{R}+\theta \tau +\alpha w=0\\ \frac{\partial H_{R}^{R}}{\partial E_{R}}=\beta (p-w)-C_{R}E_{R}=0 \end{array}\right.$$

Solving Equation set (14), we have Equation set (15):

(15) $$\left\{ \begin{array}{l} p=\frac{Q+\beta E_{R}+\theta \tau +\alpha w}{2\alpha }\\ E_{R}=\frac{\beta (p-w)}{C_{R}} \end{array}\right.$$

Substituting p from Equation set (15) into Equation (5), we can obtain the Nash equilibrium condition for the manufacturer’s Hamilton function:

(16) $$\frac{\partial H_{M}^{R}}{\partial w}=\frac{-2\alpha w+Q+\beta E_{R}+\theta \tau +\alpha c}{2}=0$$

(17) $$\frac{\partial H_{M}^{R}}{\partial E_{M}}=-C_{M}E_{M}+\gamma u_{M}^{R}=0$$

(18) $$\frac{\partial H_{M}^{R}}{\partial u_{M}^{R}}=\gamma E_{M}-\delta \tau =\tau^{\prime }$$

(19) $$u_{M}^{R}{}^{\prime }=\gamma u_{M}^{R}-\frac{\partial H_{M}^{R}}{\partial \tau }=(\gamma +\delta )u_{M}^{R}-\frac{\theta (w-c)}{2}$$

From Equation (16), we can obtain

(20) $$w=\frac{Q+\beta E_{R}+\theta \tau +\alpha c}{2\alpha }$$

Substituting Equation (20) into Equation (19), we obtain

(21) $$u_{M}^{R}{}^{\prime }=(\gamma +\delta )u_{M}^{R}-\frac{\theta (Q+\beta E_{R}+\theta \tau -\alpha c)}{4\alpha }$$

From Equation (17), we obtain

(22) $$E_{M}=\frac{\gamma u_{M}^{R}}{C_{M}}$$

Substituting Equation (22) into Equation (18), we obtain

(23) $$\tau^{\prime }=\frac{\gamma^{2}u_{M}^{R}}{C_{M}}-\delta \tau$$

If we let Equations (21) and (23) equal zero and solve them simultaneously, we obtain the stable state of the system:

$$\left(\bar{\tau_{M}^{R}},\;\bar{u_{m}^{R}})=(\frac{\theta C_{R}\gamma^{2}(Q-\alpha c)}{\delta C_{M}A(\gamma +\delta )-C_{R}\theta^{2}\gamma^{2}},\;\frac{\delta \theta C_{R}C_{M}(Q-\alpha c)}{\delta C_{M}A(\gamma +\delta )-C_{R}\theta^{2}\gamma^{2}}\right)$$

Differentiating both sides of Equation (23) with respect to t, we obtain

(24) $$\tau^{\prime \prime }=\frac{\gamma^{2}u_{M}^{R}{}^{\prime }}{C_{M}}-\delta \tau^{\prime }$$

Substituting Equation (21) into Equation (24), we obtain

(25) $$\tau^{\prime \prime }=\frac{\gamma^{2}}{C_{M}}[(\gamma +\delta )u_{M}^{R}-\frac{\theta (Q+\beta E_{R}+\theta \tau -\alpha c)}{4\alpha }]-\delta \tau^{\prime }$$

From Equation (17), we obtain

(26) $$u_{M}^{R}=\frac{C_{M}E_{M}}{\gamma }$$

Substituting Equation (26) into Equation (25) and combining with Equation (18), we obtain

(27) $$\tau^{\prime \prime }-\gamma \tau^{\prime }-[\delta (\gamma +\delta )-\frac{C_{R}\theta^{2}\gamma^{2}}{C_{M}A}]\tau =-\frac{\theta C_{R}\gamma^{2}(Q-\alpha c)}{C_{M}A}$$

Equation (27) is a second-order differential equation of τ. It is easy to determine that the equation has a positive characteristic root. To converge the solutions of the differential equation, the other characteristic root must also be negative, i.e., it is necessary to assume $\delta C_{M}A(\gamma +\delta )-C_{R}\theta^{2}\gamma^{2}>0$ in Equation (26). In addition, the initial value $\tau (0)=\tau_{0},\;\underset{t\to \infty }{\lim}\tau (t)=\bar{\tau_{M}^{R}}$. Thus, we obtain

$$\tau^{R}=(\tau_{0}-\bar{\tau_{M}^{R}})e^{-\frac{\varphi_{R}-C_{M}A\gamma }{2C_{M}A}t}+\bar{\tau_{M}^{R}}$$

Substituting into Equation (18), we obtain

$$E_{M}^{R*}=\frac{\left(2\delta C_{M}A+C_{M}A\gamma -\varphi_{R})(\tau_{0}-\bar{\tau_{M}^{R}}\right)}{2C_{M}A\gamma }e^{-\frac{\varphi_{R}-C_{M}A\gamma }{2C_{M}A}t}+\frac{\delta }{\gamma }\bar{\tau_{M}^{R}}$$

Combining Equations (15) and (16), we obtain

$$E_{R}^{R*}=\frac{\beta (Q-\alpha c)}{A}+\frac{\beta \theta }{A}[(\tau_{0}-\bar{\tau_{M}^{R}})e^{-\frac{\varphi_{R}-C_{M}A\gamma }{2C_{M}A}t}+\bar{\tau_{M}^{R}}]$$

Other optimal solutions can be similarly obtained.

Proposition 1 Q.E.D. □

According to Proposition 1, when the carbon emissions reduction per product unit is initially small, i.e.,$\tau_{0}<\bar{\tau_{M}^{R}}$, Proposition 2 can be derived as follows:

**Inference** **1.**
*(1)*

$\frac{\partial p^{R*}}{\partial t}>0,\;\frac{\partial E_{R}^{R*}}{\partial t}>0,\;\frac{\partial w^{R*}}{\partial t}>0,\;\frac{\partial q^{R*}}{\partial t}>0;$

*(2)*

$\gamma >\frac{\varphi_{R}-2\delta C_{M}A}{C_{M}A},\;\frac{\partial E_{M}^{R*}}{\partial t}>0;\;when\;\gamma <\frac{\varphi_{R}-2\delta C_{M}A}{C_{M}A},\;\frac{\partial E_{M}^{R*}}{\partial t}<0;$

*(3)*

$\frac{\partial \tau^{R}}{\partial t}>0.$

$\begin{array}{lll} (1)\; & \frac{\partial p^{R*}}{\partial t} & =-\frac{3\theta C_{R}}{2C_{M}A^{2}}(\tau_{0}-\bar{\tau_{M}^{R}})(\varphi_{R}-C_{M}A\gamma )e^{-\frac{\varphi_{R}-C_{M}A\gamma }{2C_{M}A}t}>0\\ & \frac{\partial E_{R}^{R*}}{\partial t} & =-\frac{\beta \theta }{2C_{M}A^{2}}(\tau_{0}-\bar{\tau_{M}^{R}})(\varphi_{R}-C_{M}A\gamma )e^{-\frac{\varphi_{R}-C_{M}A\gamma }{2C_{M}A}t}>0,\\ & \frac{\partial w^{R*}}{\partial t} & =-\frac{\theta C_{R}}{C_{M}A^{2}}(\tau_{0}-\bar{\tau_{M}^{R}})(\varphi_{R}-C_{M}A\gamma )e^{-\frac{\varphi_{R}-C_{M}A\gamma }{2C_{M}A}t}>0,\\ & \frac{\partial q^{R*}}{\partial t} & =-\frac{\alpha \theta C_{R}}{2C_{M}A^{2}}(\tau_{0}-\bar{\tau_{M}^{R}})(\varphi_{R}-C_{M}A\gamma )e^{-\frac{\varphi_{R}-C_{M}A\gamma }{2C_{M}A}t}>0. \end{array}$.

(2) and (3) can be similarly proven. Inference 1 Q.E.D.

Inference 1 shows that the carbon emissions reduction per product unit increases with time and gradually converges to a stable point. For manufacturers, when the impact of emissions reduction efforts on emissions reductions per product unit is greater than $\frac{\varphi_{R}-2\delta C_{M}A}{C_{M}A}$, the manufacturer’s efforts to reduce carbon emissions increases with time. When the impact of emissions reduction efforts on emissions reductions per product unit is less than $\frac{\varphi_{R}-2\delta C_{M}A}{C_{M}A}$, the manufacturer’s efforts to reduce carbon emissions decreases over time. However, the wholesale price per unit of the low-carbon product increases over time.

Therefore, the promotion of consumer awareness of low-carbon environmental protection, i.e., the promotion of low-carbon products, is important. Retailer promotional effort in support of low-carbon products increases with time, and the greater that the promotional effort is, the greater the corresponding cost. In addition, the unit wholesale price for low-carbon products increases, ultimately resulting in an increase in the retail price over time. While the retail price of low-carbon products increases, manufacturer carbon reduction efforts and retailer low-carbon product promotion contribute to the sales of these products, and the promotional effect is greater than the impact of the price increase, resulting in a low-carbon sales increase. That is, the manufacturers’ carbon emissions reduction efforts and the retailers’ low-carbon product promotion are conducive to low-carbon product sales and low-carbon industry development.

#### 3.1.2. When the Manufacturer Bears the Promotional Cost of Low-Carbon Products

Equation (28) is the manufacturer’s objective function.

(28) $$\underset{w,E_{M}}{\max}J_{M}^{M}=\int_{0}^{\infty }e^{-\rho t}[(w-c)(Q-\alpha p+\beta E_{R}+\theta \tau )-\frac{C_{M}E_{M}{}^{2}}{2}-\frac{C_{R}E_{R}{}^{2}}{2}]d(t)$$

Equation (29) is the retailer’s objective function.

(29) $$\underset{p,E_{R}}{\max}J_{R}^{M}=\int_{0}^{\infty }e^{-\rho t}[(p-w)(Q-\alpha p+\beta E_{R}+\theta \tau )]d(t)$$

The Hamilton functions of the manufacturer and the retailer are as follows:

(30) $$\begin{array}{ll} H_{M}^{M}(w,\;E_{M},\;E_{R},\;u_{M}^{M})= & \left(w-c)(Q-\alpha p+\beta E_{R}+\theta \tau \right)\\ & -\frac{C_{M}E_{M}{}^{2}}{2}-\frac{C_{R}E_{R}{}^{2}}{2}+u_{M}^{M}(\gamma E_{M}-\delta \tau ) \end{array}$$

(31) $$H_{R}^{M}(p,u_{R}^{M})=(p-w)(Q-\alpha p+\beta E_{R}+\theta \tau )+u_{R}^{M}(\gamma E_{M}-\delta \tau )$$

**Lemma** **2.** *Equation (31) is a concave function about*p, *and after substituting the optimal solution obtained from Equation (31) into Equation (30), Equation (30) is a concave function about *$w,E_{M},E_{R}$.

**Proof.**  The first- and second-order partial derivatives of Equation (31) about *p* are as follows:

(32) $$\frac{\partial H_{R}^{M}}{\partial p}=Q-2\alpha p+\beta E_{R}+\theta \tau +\alpha w,\;\frac{\partial^{2}H_{R}^{M}}{\partial p^{2}}=-2\alpha$$

from $\frac{\partial^{2}H_{R}^{M}}{\partial p^{2}}=-2\alpha <0$, we know that Equation (31) is a concave function about p. Then, from $\frac{\partial H_{R}^{M}}{\partial p}=Q-2\alpha p+\beta E_{R}+\theta \tau +\alpha w=0$, we obtain

(33) $$p=\frac{Q+\beta E_{R}+\theta \tau +\alpha w}{2\alpha }$$

Substituting Equation (32) into Equation (30) and rearranging, we obtain

(34) $$H_{M}^{M}=\frac{\left(w-c)(Q+\beta E_{R}+\theta \tau -\alpha w\right)}{2}-\frac{C_{M}E_{M}{}^{2}}{2}-\frac{C_{R}E_{R}{}^{2}}{2}+u_{M}^{M}(\gamma E_{M}-\delta \tau )$$

The Hessian matrix of Equation (34) is as follows:

(35) $$H=\left[\begin{array}{ccc} \frac{\partial^{2}H_{M}^{M}}{\partial w^{2}} & \frac{\partial^{2}H_{M}^{M}}{\partial w\partial E_{R}} & \frac{\partial^{2}H_{M}^{M}}{\partial w\partial E_{M}}\\ \frac{\partial^{2}H_{M}^{M}}{\partial E_{R}\partial w} & \frac{\partial^{2}H_{M}^{M}}{\partial E_{R}{}^{2}} & \frac{\partial^{2}H_{M}^{M}}{\partial E_{R}\partial E_{M}}\\ \frac{\partial^{2}H_{M}^{M}}{\partial E_{M}\partial w} & \frac{\partial^{2}H_{M}^{M}}{\partial E_{M}\partial E_{R}} & \frac{\partial^{2}H_{M}^{M}}{\partial E_{M}{}^{2}} \end{array}\right]=\left[\begin{array}{ccc} -2\alpha & \frac{\beta }{2} & 0\\ \frac{\beta }{2} & -C_{R} & 0\\ 0 & 0 & -1 \end{array}\right]$$

From Equation (35), we know that all the second-order determinants of matrix H are greater than zero, the third-order matrix is less than zero, and the diagonal elements are all less than zero. Therefore, Equation (30) is a concave function about $w,E_{M},E_{R}$. Under the guarantee of Lemma 2, Proposition 2 can be obtained as follows. □

**Proposition** **2.** *The steady state of the system is as follows:*

(36) $$\left(\bar{\tau_{M}^{M}},\;\bar{u_{m}^{M}})=(\frac{2\theta C_{R}\gamma^{2}(Q-\alpha c)}{\delta C_{M}A(\gamma +\delta )-2C_{R}\theta^{2}\gamma^{2}},\;\frac{2\delta \theta C_{R}C_{M}(Q-\alpha c)}{\delta C_{M}A(\gamma +\delta )-2C_{R}\theta^{2}\gamma^{2}}\right)$$

*The retailer’s optimal unit retail price and sales volume are as follows:*

(37) $$P^{M*}=\frac{3QC_{R}+c(\alpha C_{R}-\beta^{2})}{A}+\frac{3\theta C_{R}}{A}[(\tau_{0}-\bar{\tau_{M}^{M}})e^{-\frac{\varphi_{M}-C_{M}B\gamma }{2C_{M}B}t}+\bar{\tau_{M}^{M}}]$$

(38) $$q^{M*}=\frac{\alpha C_{R}(Q-\alpha c)}{A}+\frac{\alpha \theta C_{R}}{A}[(\tau_{0}-\bar{\tau_{M}^{M}})e^{-\frac{\varphi_{M}-B\gamma }{2B}t}+\bar{\tau_{M}^{M}}]$$

*The manufacturer’s optimal unit wholesale price, the degree of emissions reduction efforts, and the degree of promotional efforts are as follows, respectively:*

(39) $$w^{M*}=\frac{2C_{R}Q+c(2\alpha C_{R}-\beta^{2})}{A}+\frac{2\theta C_{R}}{A}[(\tau_{0}-\bar{\tau_{M}^{M}})e^{-\frac{\varphi_{M}-C_{M}A\gamma }{2C_{M}A}t}+\bar{\tau_{M}^{M}}]$$

(40) $$E_{M}^{M*}=\frac{\left(2\delta C_{M}A+C_{M}A\gamma -\varphi_{M})(\tau_{0}-\bar{\tau_{M}^{M}}\right)}{2C_{M}A\gamma }e^{-\frac{\varphi_{M}-C_{M}A\gamma }{2C_{M}A}t}+\frac{\delta }{\gamma }\bar{\tau_{M}^{M}}$$

(41) $$E_{R}^{M*}=\frac{\beta (Q-\alpha c)}{A}+\frac{\beta \theta }{A}[(\tau_{0}-\bar{\tau_{M}^{M}})e^{-\frac{\varphi_{M}-C_{M}A\gamma }{2C_{M}A}t}+\bar{\tau_{M}^{M}}]$$

*The optimal trajectory of the manufacturer’s product emissions reduction is as follows:*

(42) $$\tau^{M}=(\tau_{0}-\bar{\tau_{M}^{M}})e^{-\frac{\varphi_{M}-C_{M}A\gamma }{2C_{M}A}t}+\bar{\tau_{M}^{M}}$$

*where*$\varphi_{M}=\sqrt{AC_{M}[AC_{M}{\left(\gamma +2\delta \right)}^{2}-8C_{R}\theta^{2}\gamma^{2}]}$.

The proof of Proposition 2 is similar to that of Proposition 1 and the proof of Proposition 2 is omitted here.

According to Proposition 2, when the carbon emissions reduction per product unit is initially small, i.e., $\tau_{0}<\bar{\tau_{M}^{M}}$, Inference 2 can be derived as follows:

**Inference** **2.**
*(1)*

$\frac{\partial p^{M*}}{\partial t}>0,\;\frac{\partial E_{R}^{M*}}{\partial t}>0,\;\frac{\partial w^{M*}}{\partial t}>0,\;\frac{\partial q^{M*}}{\partial t}>0;$

*(2)*

$when\;\gamma >\frac{\varphi_{M}-2\delta C_{M}A}{C_{M}A},\;\frac{\partial E_{M}^{M*}}{\partial t}>0;\;when\;\gamma <\frac{\varphi_{M}-2\delta C_{M}A}{C_{M}A}\;,\;\frac{\partial E_{M}^{M*}}{\partial t}<0;$

*(3)*

$\frac{\partial \tau^{M}}{\partial t}>0.$

The proof of Inference 2 is similar with that of Inference 1, so it is omitted here.

Inference 2 shows that the unit whole price, the unit retail price, the sales quantity, and the amount of emissions reduction per product unit increase with time. Moreover, the low-carbon product promotion effort also increases with time when the manufacturer bears the promotional cost of low-carbon products. However, the manufacturer’s efforts to reduce carbon emissions increase with time when the impact of emissions reduction efforts on emissions reductions per product unit is greater than $\frac{\varphi_{M}-2\delta C_{M}A}{C_{M}A}$, otherwise, the manufacturer’s efforts to reduce carbon emissions decrease over time.

Based on the Inference 1 and Inference 2, the unit whole price, the unit retail price, the sales quantity, and the amount of emissions reduction per product unit increase with time regardless of who will bear the promotional cost of low-carbon products. There are two reasons: one is that consumer awareness of environmental protection is growing, so the manufacturers have to decrease the amount of emissions; secondly, although the retail price increases, the sales volume often increases, because consumers are willing to pay more for products with less carbon emissions.

### 3.2. Under Centralized Decision-Making

(43) $$\underset{p,E_{M},E_{R}}{\max}J_{C}=\int_{0}^{\infty }e^{-\rho t}[(p-c)(Q-\alpha p+\beta E_{R}+\theta \tau )-\frac{C_{M}E_{M}{}^{2}}{2}-\frac{C_{R}E_{R}{}^{2}}{2}]d(t)$$

The Hamilton function of Equation (44) is as follows:

(44) $$\begin{array}{ll} H^{C}(p,E_{R},E_{M},u^{C}) & =(p-c)(Q-\alpha p+\beta E_{R}+\theta \tau )\\ & -\frac{C_{M}E_{M}{}^{2}}{2}-\frac{C_{R}E_{R}{}^{2}}{2}+u^{C}(\gamma E_{M}-\delta \tau ) \end{array}$$

We obtain Lemma 3 similarly to the manner in which we obtained Lemma 2.

**Lemma** **3.** *Equation* *(44) is a concave function about* $p,E_{R},E_{M}.$

The proof of Lemma 3 is similar to that of Lemma 2 and is thus omitted here. Under the guarantee of Lemma 3, we obtain Proposition 3 as follows.

**Proposition** **3.** *The steady state of the system is as follows:*

(45) $$\left(\bar{\tau^{C}},\;\bar{u^{C}})=(\frac{\theta C_{R}\gamma^{2}(Q-\alpha c)}{\delta C_{M}B(\gamma +\delta )-C_{R}\theta^{2}\gamma^{2}},\;\frac{\delta \theta C_{R}C_{M}(Q-\alpha c)}{\delta C_{M}B(\gamma +\delta )-C_{R}\theta^{2}\gamma^{2}}\right)$$

*The optimal unit retail price and sales volume are as follows, respectively:*

(46) $$P^{C*}=\frac{QC_{R}+c(\alpha C_{R}-\beta^{2})}{B}+\frac{\theta C_{R}}{B}[(\tau_{0}-\bar{\tau^{C}})e^{-\frac{\varphi_{C}-C_{M}B\gamma }{2C_{M}B}t}+\bar{\tau^{C}}]$$

(47) $$q^{C*}=\frac{\alpha C_{R}(Q-\alpha c)}{B}+\frac{\alpha \theta C_{R}}{B}[(\tau_{0}-\bar{\tau^{C}})e^{-\frac{\varphi_{C}-BC_{M}\gamma }{2LC_{M}}t}+\bar{\tau^{C}}]$$

*The optimal level of efforts to reduce emissions and the level of promotional effort are as follows, respectively:*

(48) $$E_{M}^{C*}=\frac{\left(2\delta C_{M}B+C_{M}B\gamma -\varphi_{C})(\tau_{0}-\bar{\tau^{C}}\right)}{2C_{M}B\gamma }e^{-\frac{\varphi_{C}-C_{M}B\gamma }{2C_{M}B}t}+\frac{\delta }{\gamma }\bar{\tau^{C}}$$

(49) $$E_{R}^{C*}=\frac{\beta (Q-\alpha c)}{B}+\frac{\beta \theta }{B}[(\tau_{0}-\bar{\tau^{C}})e^{-\frac{\varphi_{C}-C_{M}B\gamma }{2C_{M}B}t}+\bar{\tau^{C}}]$$

*The optimal trajectory of emissions reduction per unit product is as follows:*

(50) $$\tau^{C}=(\tau_{0}-\bar{\tau^{C}})e^{-\frac{\varphi_{C}-C_{M}B\gamma }{2C_{M}B}t}+\bar{\tau^{C}}$$

*where*$B=2\alpha C_{R}-\beta^{2},\;\varphi_{C}=\sqrt{C_{M}B[C_{M}B{\left(\gamma +2\delta \right)}^{2}-4C_{R}\theta^{2}\gamma^{2}]}$.

According to Proposition 3, when the carbon emissions reduction per product unit is initially small, i.e., $\tau_{0}<\bar{\tau^{C}}$, Inference 3 can be derived as follows:

**Inference** **3.**
*(1)*

$\frac{\partial p^{C*}}{\partial t}>0,\;\frac{\partial E_{R}^{C*}}{\partial t}>0,\;\frac{\partial q^{C*}}{\partial t}>0;$

*(2)*

$when\;\gamma >\frac{\varphi_{C}-2\delta C_{M}B}{C_{M}B},\frac{\partial E_{M}^{C*}}{\partial t}>0;\;when\;\gamma <\frac{\varphi_{C}-2\delta C_{M}B}{C_{M}B},\;\frac{\partial E_{M}^{C*}}{\partial t}<0;$

*(3)*

$\frac{\partial \tau^{C}}{\partial t}>0$

The proof of Inference 3 is similar with that of Inference 1, so it is omitted here.

Inference 3 shows that the unit retail price, the sales quantity, and the amount of emissions reduction per product increase with time. Moreover, the low-carbon product promotion effort also increases with time when the manufacturer bears the promotional cost of low-carbon products. However, the manufacturer’s efforts to reduce carbon emissions increase with time when the impact of emissions reduction efforts on emissions reductions per product unit is greater than $\frac{\varphi_{C}-2\delta C_{M}B}{C_{M}B}$, otherwise, the manufacturer’s efforts to reduce carbon emissions decrease over time.

## 4. Comparative Analysis

**Figure 1:**
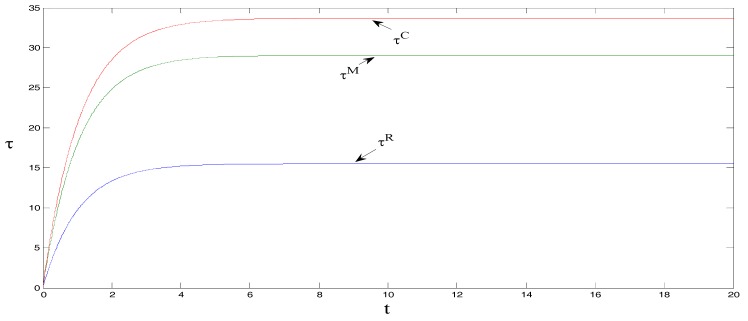
Comparison of carbon emissions reduction trajectories per product unit.

**Figure 2:**
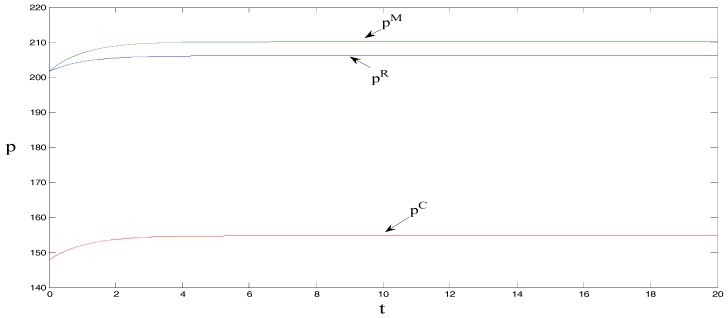
Comparison of retail prices.

**Figure 3:**
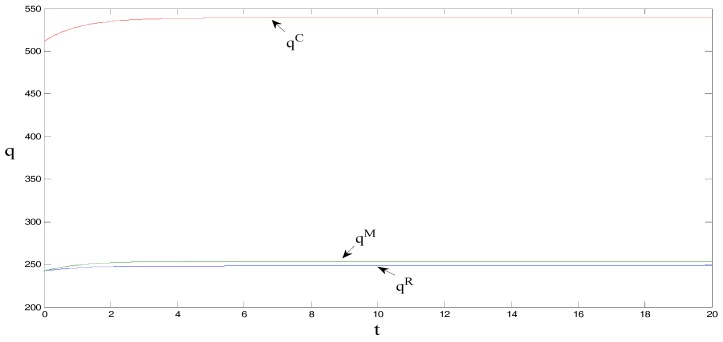
Comparison of market demand.

**Figure 4:**
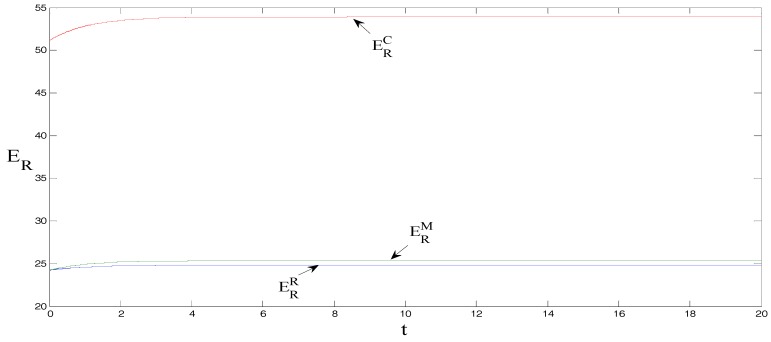
Comparison of promotional efforts.

**Figure 5:**
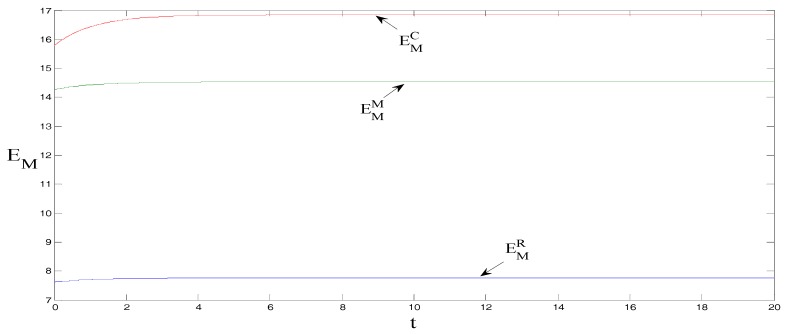
Comparison of emissions reduction efforts.

This section analyzes the impact of the different ways of bearing the promotional cost of low-carbon products and centralized decision-making in the supply chain on the stability of the system (i.e., when t→∞). The numerical simulation is carried out using MATLAB R2014a to show Inference 2 and Inference 3. Similar to [17], let Q=1000, α=4, β=2,  δ=1, θ=1.5, γ=2, $\rho =0.7,\;C_{M}=8$, and $C_{R}=5,\;\tau_{0}=0$. These initial values do not change the equilibrium findings, although they may change the initial positions in Figure 1, Figure 2, Figure 3, Figure 4 and Figure 5 [18].

### 4.1. The Impact on System Stability

**Inference** **4.** *The impact* *on system stability* *is as follows:* $(\bar{\tau_{M}^{R}},\;\bar{u_{m}^{R}})<(\bar{\tau_{M}^{M}},\;\bar{u_{m}^{M}})<(\bar{\tau^{C}},\;\bar{u^{C}}).$

**Proof.**  From $\bar{\tau_{M}^{M}}-\bar{\tau_{M}^{R}}=\frac{\delta \theta C_{R}C_{M}A^{2}\gamma^{2}(\gamma +\delta )(Q-\alpha c)}{\left[\delta C_{M}A(\gamma +\delta )-2C_{R}\theta^{2}\gamma^{2}][\delta C_{M}A(\gamma +\delta )-C_{R}\theta^{2}\gamma^{2}\right]}>0$ and $\bar{\tau^{C}}-\bar{\tau_{M}^{M}}=\frac{\delta \theta C_{R}C_{M}\beta^{2}\gamma^{2}(\gamma +\delta )(Q-\alpha c)}{\left[\delta C_{M}B(\gamma +\delta )-C_{R}\theta^{2}\gamma^{2}][\delta C_{M}A(\gamma +\delta )-2C_{R}\theta^{2}\gamma^{2}\right]}>0$, we obtain $\bar{\tau_{M}^{R}}<\bar{\tau_{M}^{M}}<\bar{\tau^{C}}.$ □

Inference 4 shows that when retailers bear the promotional costs of low-carbon products, the carbon emissions reduction per product unit is the smallest, followed by when manufacturers bear the promotional cost and lastly when under centralized decision-making. This outcome is primarily due to the fact that when the low-carbon product promotional cost is borne by retailers, their sales costs increase, causing them to decrease their promotional efforts and ultimately resulting in lower sales of low-carbon products compared with the other two scenarios. A reduction in the sales volume of low-carbon products decreases the carbon emissions reduction efforts of manufacturers and ultimately results in a decrease in carbon emissions reduction per product unit.

As shown in Figure 1, under centralized decision-making, the carbon emissions reduction trajectory per product unit is the greatest, followed by when manufacturers bear the promotional cost of low-carbon products, and lastly when retailers bear the promotional cost. The main reason is that under centralized decision-making, the sales volume of low-carbon products, promotional efforts, and carbon emissions reduction efforts per product unit are the highest. This outcome increases the willingness of the manufacturers to reduce carbon emissions for low-carbon products and ultimately promotes carbon emissions reductions, thus achieving the largest carbon emissions reduction trajectory.

### 4.2. The Impact on Price and Sales Volume

**Inference** **5.** *The impacton price and sales volume is as follows:*$p_{m}^{R*}<p_{m}^{M*}$,$q^{R}<q^{M}<q^{C}$.

The proof is similar to the proof of Inference 4, so the proof of Inference 5 is not given.

As can be observed from Figure 2 and Figure 3, the retail price per product unit is the largest when manufacturers bear the promotional cost for low-carbon products, followed by when retailers bear the cost, and lastly when under centralized decision-making. Sales volume is the greatest under centralized decision-making, followed by when manufacturers bear the promotional cost, and lastly when retailers bear the promotional cost. Regarded in combination with Figure 1 and Figure 2, the figures show that when manufacturers bear the promotional cost, the unit retail price is the highest (which results in a decrease in the sales volume of low-carbon products) but promotional efforts and carbon emissions reduction efforts per product unit are greater than when retailers bear the promotional cost. This outcome in turn promotes the sales of low-carbon products, and its impact on sales is greater than the impact of higher prices, which ultimately increases the sales volume of low-carbon products. Further, it can be noted that under centralized decision-making, the unit retail price of low-carbon products is the lowest. However, the promotional efforts and carbon reduction efforts are the highest, ultimately maximizing the sales volume. That is, the entire supply chain is optimal under centralized decision-making.

### 4.3. The Impact on Efforts

**Inference** **6.** *The impact on the manufacturers’ carbon emissions reduction efforts, the retailers’ low-carbon product promotion efforts is as follows:* $E_{M}^{R}<E_{M}^{M}<E_{M}{}^{C},\;E_{R}^{R}<E_{R}^{M}<E_{R}^{C}\;.$

The proof of Inference 6 is similar to that of Inference 2 and thus is omitted here.

Inference 6 shows that carbon emission reduction efforts and low-carbon product promotional efforts are the greatest under centralized supply-chain decision-making, followed by when the low-carbon product promotional costs are borne by manufacturers, and lastly when the low-carbon product promotional costs are borne by retailers. When the low-carbon product promotional costs are borne by manufacturers, the unit wholesale price and retail price are greater than when the low-carbon product promotional costs are borne by retailers. However, combining with Inference 2 and Inference 3, we know that the sales volume is greater when the low-carbon product promotional costs are borne by manufacturers than by retailers. Therefore, when the supply chain is not coordinated, manufacturers bearing the promotional cost of low-carbon products is conducive to the implementation of low-carbon product promotion and carbon emissions reduction.

It can be observed from Figure 4 and Figure 5 that promotional efforts in support of low-carbon products and carbon emissions reduction efforts are the largest under centralized decision-making, followed by when manufacturers bear the low-carbon product promotional costs, and lastly when retailers bear the promotional costs. Which party bears the low-carbon product promotional costs has little impact on the promotional effort but significantly affects carbon emissions reduction efforts per product unit. This phenomenon primarily occurs because when the manufacturers bear the promotional cost of low-carbon products, the level of carbon emissions reduction efforts increases, which is more conducive to the promotion of low-carbon products. Therefore, when manufacturers bear the promotional costs of low-carbon products, the carbon emissions reduction effort per product unit is significantly greater than when retailers bear the promotional costs.

## 5. Conclusions

This paper constructs a derivative game model for manufacturers and retailers. We use the model to analyze the impact of emissions reduction efforts and low-carbon product promotion on the supply-chain carbon emissions reduction strategy under three scenarios: (1) when the low-carbon product promotional cost is borne by either the manufacturers or (2) the retailers and (3) under centralized decision-making in the supply chain. The main conclusions are as follows:

(1) The retail price per product unit increases when manufacturers bear the promotional cost of low-carbon products. However, the corresponding carbon reduction effort and promotional effort are greater than when retailers bear the promotional cost. In addition, the impact of carbon emissions reduction and low-carbon product promotion on the demand for low-carbon products is greater than the impact of increased prices on sales, ultimately promoting low-carbon product sales volume. Not only can the retail price per unit product be decreased, but the emissions reduction efforts and promotional efforts can also be increased to promote low-carbon sales when the supply chain is under centralized decision-making. That is, centralized decision-making in the supply chain can optimize the supply chain.
(2) The carbon emissions reduction per product unit is the smallest when retailers bear the promotion costs, followed by when manufacturers bear the promotional cost, and lastly when under centralized decision-making. The main reason is that under centralized decision-making, the sales volume of low-carbon products, promotional efforts, and carbon emissions reduction efforts per product unit are the highest. This outcome increases the willingness of the manufacturers to reduce carbon emissions for low-carbon products and ultimately promotes carbon emissions reductions, thus achieving the largest carbon emissions reduction trajectory. Promotional efforts in support of low-carbon products and carbon emissions reduction efforts are the largest under centralized decision-making, followed by when manufacturers bear the promotional costs, and lastly when retailers bear the promotional costs. This phenomenon primarily occurs because when the manufacturers bear the promotional cost of low-carbon products, the level of carbon emissions reduction efforts increases, which is more conducive to the promotion of low-carbon products.
(3) The retail price per product unit is the largest when manufacturers bear the promotional cost for low-carbon products, followed by when retailers bear the cost, and lastly when under centralized decision-making. Sales volume is the greatest under centralized decision-making, followed by when manufacturers bear the promotional cost, and lastly when retailers bear the promotional cost. This outcome in turn promotes the sales of low-carbon products, and its impact on sales is greater than the impact of higher prices, which ultimately increases the sales volume of low-carbon products. Further, it can be noted that under centralized decision-making, the unit retail price of low-carbon products is the lowest. However, the promotional efforts and carbon reduction efforts are the highest, ultimately maximizing the sales volume. That is, the entire supply chain is optimal under centralized decision-making.
